# Quantitative Analysis of Collective Migration by Single-Cell Tracking Aimed at Understanding Cancer Metastasis

**DOI:** 10.3390/ijms232012372

**Published:** 2022-10-15

**Authors:** Zhuohan Xin, Keiko Deguchi, Shin-ichiro Suye, Satoshi Fujita

**Affiliations:** 1 Department of Advanced Interdisciplinary Science and Technology, University of Fukui, Fukui 910-8507, Japan; 2 Department of Frontier Fiber Technology and Science, University of Fukui, Fukui 910-8507, Japan; 3 Organization for Life Science Advancement Programs, University of Fukui, Fukui 910-8507, Japan

**Keywords:** collective migration, epithelial–mesenchymal transition, electrospinning, cell interaction, metastasis

## Abstract

Metastasis is a major complication of cancer treatments. Studies of the migratory behavior of cells are needed to investigate and control metastasis. Metastasis is based on the epithelial–mesenchymal transition, in which epithelial cells acquire mesenchymal properties and the ability to leave the population to invade other regions of the body. In collective migration, highly migratory “leader” cells are found at the front of the cell population, as well as cells that “follow” these leader cells. However, the interactions between these cells are not well understood. We examined the migration properties of leader–follower cells during collective migration at the single-cell level. Different mixed ratios of “leader” and “follower” cell populations were compared. Collective migration was quantitatively analyzed from two perspectives: cell migration within the colony and migration of the entire colony. Analysis of the effect of the cell mixing ratio on migration behavior showed that a small number of highly migratory cells enhanced some of the migratory properties of other cells. The results provide useful insights into the cellular interactions in collective cell migration of cancer cell invasion.

## 1. Introduction

Cancer cells possess the ability to metastasize, a multi-step regulated process that involves leaving the primary tumor site, invading the mesenchyme, circulating in the blood or lymphatic vessels, and invading distant organs through extravasation. Metastasis creates major challenges in cancer treatment. Before metastasis, early-stage cancers can be treated locally using surgery or radiotherapy. However, after cancer has metastasized and spread, it is difficult to completely cure this disease [1]. Therefore, studies are needed to understand the mechanisms underlying cancer metastasis to facilitate cancer treatment.

The basic mechanisms of cell migration have been extensively studied. Cell migration is based on the establishment of a front-to-rear polarity axis involving rearrangement of the cytoskeleton and polarization of the membranes. Underlying this front-to-rear polarity is front-to-rear polarization of the Rho family signaling cascade, whereby Rac1 and CDC42 induce rapid cytoskeletal rearrangements at the front of a cell [2]. This leads to the formation of membrane-like protrusions such as filopodia and lamellipodia. Adhesion between the cell adhesion protein integrin and the extracellular matrix (ECM) is promoted, leading to forward migration of cells [3]. However, during collective migration, cells invade the ECM while maintaining E-cadherin-dependent cell adhesion. During collective migration, the migration mechanism of individual cells occurs for each cell in the population. In addition, there is a leader cell with a highly invasive and ECM remodeling capacity in collective migration and subsequent group of follower cells [4]. The leader and follower cells are defined based only on their relative positions in the cluster and are located at the front and rear of the cluster, respectively. Leader cells are highly invasive and play important roles in ECM remodeling during migration [5].

In the early stages of cancer metastasis, cancer cells leave the primary tumor as individual cells and migrate through the effects of epithelial–mesenchymal transition (EMT). EMT involves a series of biological changes in which epithelial cells lose their epithelial properties and acquire mesenchymal properties. When EMT is induced in cells, the cell function and characteristics are greatly altered; such changes include loss of cell polarity, altered cell morphology, and acquisition of invasive capacity, along with downregulation of epithelial cell genes and upregulation of mesenchymal cell genes [6,7]. Transforming growth factor-β (TGF-β) is a cell growth factor and representative EMT-inducing factor [8]. When TGF-β acts on epithelial cells, various transcriptional regulatory mechanisms are activated through the TGF-β signaling pathway. TGF-β induces EMT by inhibiting the transcription of intercellular adhesion molecules (e.g., E-cadherin), decreasing genes characteristically expressed in epithelial cells, and increasing genes characteristically expressed in mesenchymal cells [9]. In EMT, epithelial and mesenchymal features are considered as binary “on/off.” However, in vitro experiments showed that epithelial and mesenchymal markers are coexpressed in the same cells [10]. This observation suggests that EMT progressively develops in a state in which epithelial and mesenchymal properties are mixed [11]. Screening for cell surface markers in breast cancer showed that EMT exhibits a distinctive mixed phenotype and develops in a progressive pattern [12]. Furthermore, these mixed tumor cell subpopulations increase the metastatic potential in vivo [13]. This diverse mixed phenotype is involved in regulating collective migration and forming a “leader–follower” structure at different stages of EMT [14]. These results suggest that EMT-induced cells behave as “leaders,” exhibiting migratory behavior along fibrous structures in the ECM. However, the interactions between “leader” and “follower” cells and role of migration enhancement in metastasis remain unclear.

The microenvironment surrounding the tissues plays an important role in maintaining normal cellular behavior. The microenvironment varies between normal tissues and tumors, suggesting that cancer development and metastasis are influenced by the surrounding microenvironment [15,16]. Classical cell migration assays, such as chemotaxis assays [17,18], involve the addition of chemokines to the culture environment to induce cell migration according to a concentration gradient. Wound healing assays [19,20] based on scratch assays can be used to evaluate cell migration properties by measuring tissue matrix build-up and associated cell differences in healing. In chemotaxis assays, environments with fixed concentration gradients are uncommon in cancer cell migration in vivo; the manner in which cells migrate in scratch assays differs from that in which cancer cells migrate collectively in a 3D environment. Traditional assays cannot adequately track cell population migration, supporting the necessity of constructing cancer cell migration models that simulate the in vivo environment. A recently established migration evaluation system mimics the in vivo cellular environment. In this system, cells migrate on flat culture dishes coated with fibronectin (FN) or ECM gels (e.g., collagen) present in the ECM [21]. Cells present in the ECM in a fibrous structure have an elongated morphology in vivo. Therefore, in flat culture dishes or gels without anisotropy, cells have a different morphology and applied tension compared to their original morphology. The extension and migration directions of pseudopods, which are important for cell migration, may greatly differ from the original morphology [22]. Nanofiber materials fabricated by electrospinning have attracted considerable attention. Because nanofibers mimic fibrous and anisotropic structures in vivo, they are expected to be used as scaffolds in regenerative medicine [23,24].

In this study, we focused on leader–follower cell interactions. To quantitatively analyze these interactions and reproducibly observe cell migration on fibers, we designed a method to mimic collective migration by co-culturing TGF-β1-induced EMT mesenchymal cells [TGF (+)] with EMT-negative cells [TGF (−)]. We evaluated the migration capacity of mimicked cell populations. We generated populations of TGF (+) and TGF (−) cells at different ratios and compared their migratory behaviors. The morphology, trajectory, migration velocity, straightness, and directional angle were evaluated to quantitatively examine the migration of cell populations and effect of TGF (+) on cell migration.

## 2. Results

### 2.1. Cell Migration on Fiber

**Figure 1:**
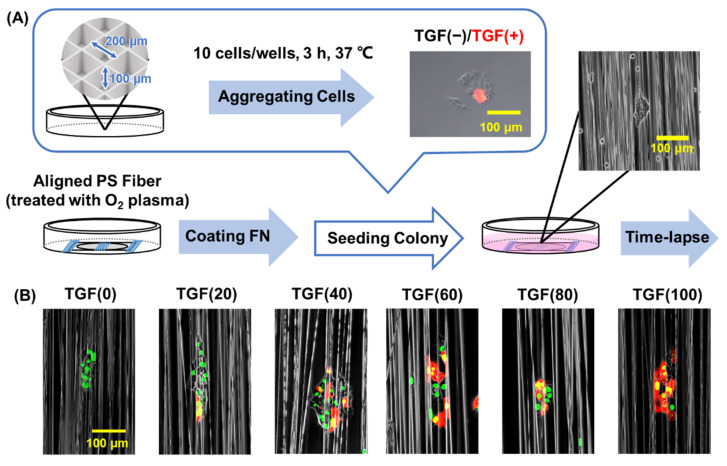
Experimental procedure. (**A**) Schematic diagram of the preparation of cell aggregate and seeding onto aligned polystyrene (PS) fibers. (**B**) Aggregates of transforming growth factor (TGF) (+) cells (red) and TGF (−) cells (green) were inoculated onto the fibers.

We characterized the motility of migrating cells in a cell population by analyzing cell behaviors at the single-cell level. The migration of cell populations with different ratios of mesenchymal and epithelial cells was analyzed. First, we set up a system to observe collective migration using different ratios of TGF (+) and TGF (−) cells on directed polystyrene (PS) fibers, the surfaces of which were coated with ECM protein (FN). This design mimics the fibrous structure in vivo (Figure 1A). Colony elongation and migration of TGF (+) and TGF (−) cells were evaluated as the migration of cell colonies at different TGF (+) mixing ratios (Figure 1B, Movies S1–S6). TGF (+) cells were induced with NMuMG-expressing DsRed to distinguish TGF (+) from TGF (−). Under different percentages of TGF (+) cells, the cells migrated in the colony within 24 h. In the absence of TGF (+) cells (TGF (0)), the cells were strongly attached to each other, and the shape of the population did not change significantly. When the percentage of TGF (+) exceeded 50%, the cells were loosely adhered and frequently separated from the colony.

### 2.2. Colony Migration

**Figure 2:**
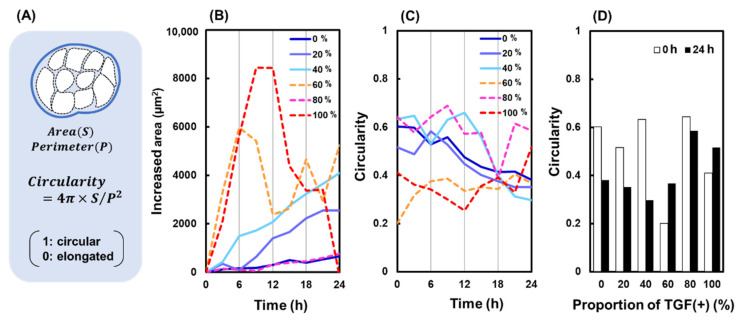
Changes in colony shape. (**A**) Definition of circularity. (**B**) Area at different TGF (N) and time. (**C**) Circularity changes over different times. (**D**) Circularity at 0 and 24 h with different TGF (N).

The shapes of cell colonies with different TGF (+)/TGF (−) ratios during migration are shown in Figure 2B. In TGF (0)–(40), in which TGF (+) cells accounted for the minority of cells, the area increased as N increased and with increasing observation time. Figure 2C shows the variation in circularity over time, and Figure 2D shows a comparison of circularity at 0 and 24 h. In TGF (0)–(40) cells, the circularity at 24 h decreased significantly compared to that at 0 h, indicating an elongated colony shape. In contrast, in TGF (60)–(100), changes in the area and circularity revealed an unstable morphology, possibly because of the mesenchymal properties of TGF (+) in most of the colonies, leading to weak cell adhesion and more dispersed migration.

**Figure 3:**
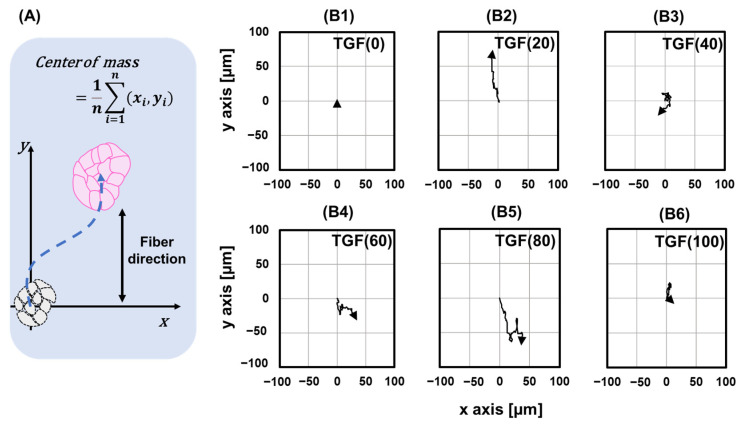
Changes in the location of the colony center. (**A**) Definition of center of mass. (**B1**–**B6**) Movement of center of mass under different TGF (N).

In Figure 3, TGF (0) and TGF (100) did not move as colonies, as their endpoints returned to their original state. TGF (20) moved along the fiber, showing only slight changes in the direction of movement. TGF (80) also moved approximately the same distance but in repeated forward and backward directions. The centers of mass of TGF (40) and TGF (60) moved similar distances.

### 2.3. Migration of Single Cells

#### 2.3.1. Changes in Cell Trajectories

**Figure 4:**
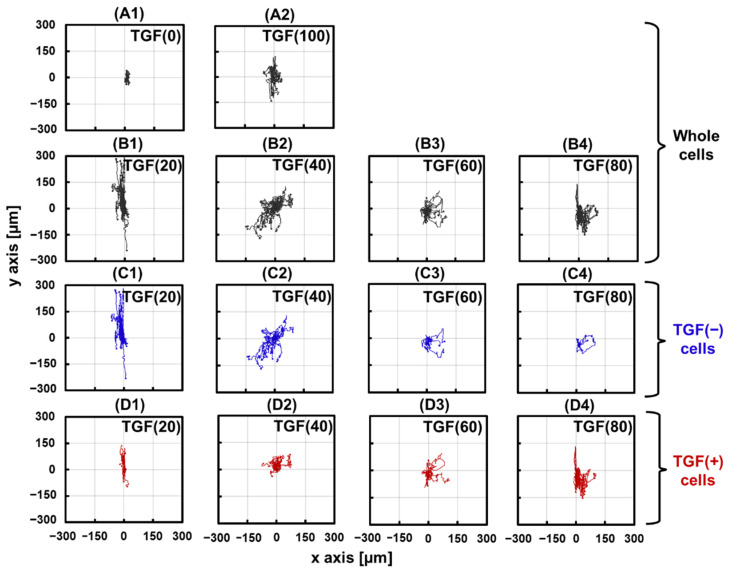
Changes in cell trajectories. (**A1**,**A2**) and (**B1**–**B4**) trajectories of TGF (+) and TGF (−) under different TGF (N). (**C1**–**C4**) Trajectories of TGF (−) cells. (**D1**–**D4**) Trajectories of TGF (+) cells.

Next, the trajectories of the cells were plotted by extracting the time-series data of the coordinates of the nuclei. The starting point of all cell trajectories was reset to the origin (0,0), as shown in Figure 4. In TGF (0) cells (Figure 4(A1)), the trajectory did not spread, indicating limited cell migration. In TGF (20) (Figure 4(B1)), the cells migrated mainly along the fibers (*y*-axis direction). In TGF (40)–(80), the cells migrated along the direction of the fibers and spread in the *x*-axis direction (Figure 4(B2–B4)). We plotted the trajectories of TGF (+) and TGF (−) separately to investigate migration of these populations independently (Figure 4C1–C4,D1–D4). TGF (−) cells showed greater migration compared to that of TGF (0) cells under all conditions. In addition, TGF (+) and TGF (−) cells belonging to the same colony exhibited similar trajectories in the migration direction. These results suggest that TGF (+) and TGF (−) interact in the same colony and alter the migratory behavior of the entire colony. Interestingly, TGF (−) showed higher migration in TGF (20) and TGF (40) than in TGF (+) (Figure 4(C1,C2,D1,D2)). This result suggests that the presence of a minority of TGF (+) cells in the colony enhances TGF (−) migration.

#### 2.3.2. Changes in Straightness and Velocity

**Figure 5:**
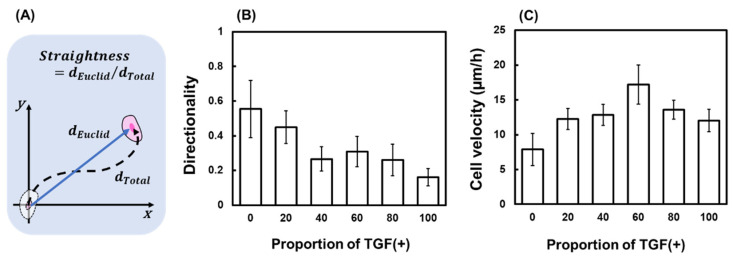
Changes in migration direction and velocity. (**A**) Definition of straightness. (**B**) Mean straightness of all cells with different TGF (N). (**C**) Mean velocity of all cells with different TGF (N).

In TGF (0), the straightness was 0.55 ± 0.17 μm but decreased with increasing N, reaching a minimum value of 0.16 ± 0.05 μm in TGF (100) (Figure 5B). For TGF (0), intercellular adhesion was strong, and almost no migration was observed. With increasing N, intercellular adhesion loosened, possibly because of a large change in direction related to the increased migratory capacity of each cell. The minimum value of the average migration velocity of the cells was obtained under the TGF (0) condition (7.89 ± 2.33 µm/h). The maximum value was obtained under the TGF (60) condition (17.10 ± 2.31 µm/h) (Figure 5C).

#### 2.3.3. Changes in Direction Angle

**Figure 6:**
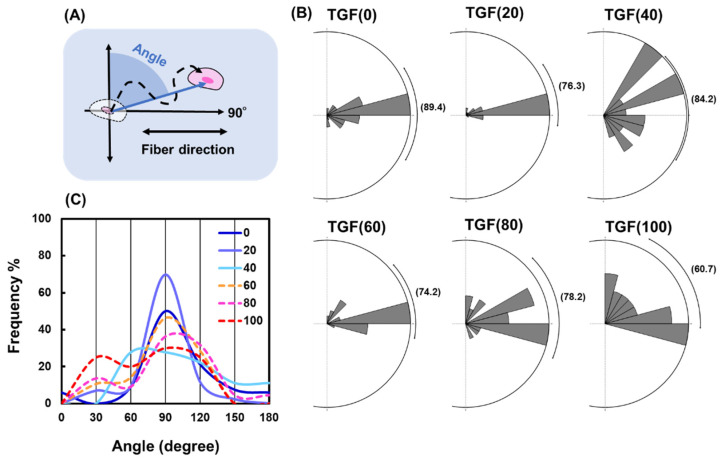
Changes in direction angle. (**A**) Definition of direction angle. (**B**) Distribution of direction angles and mean values with different TGF (N). (**C**) Frequency of direction angle values with different TGF (N).

Straightness does not represent the directionality of cell migration. Therefore, the directional angle during cell migration was calculated and displayed as a rose plot (Figure 6B). The directional angle is the angle between the direction of cell migration and direction perpendicular to the fiber (Figure 6A). The mean value of TGF (0) was closest to 90°; as N increased, this value differed from 90°, indicating that when the ratio of TGF (+) is higher, fewer cells in the colony migrate along the fibers. Figure 6C shows a histogram of the direction angle for each condition; as N decreased, a clear peak was formed at 90°. This decrease in variability clearly indicates that a change in the ratio of TGF (+) to TGF (−) alters the migration of the cell population.

## 3. Discussion

We examined how the presence of TGF (+) affects each cell in a mixed colony in different situations. In the first situation, N < 50. When N = 20, TGF (−) exhibited a trajectory along the fiber direction (Figure 4(C1)). When N = 40, the migration of TGF (−) was enhanced not only in the fiber direction, but also in other directions (Figure 4(C2)). This pattern was also observed for larger N, which corresponds to the results (Figure 4(C3,C4)). Similar effects were observed not only in the distance of migration, but also in velocity (Figure 5C). The velocity of TGF (−) increased with increasing N at N < 50. Under this condition, there was more TGF (−), and TGF (+) may have enhanced TGF (−) migration as N increased. This observation is similar to the relationship between leaders and their followers. In collective invasion, leader cells express basal epithelial genes such as cytokeratin-14 and p63 [25]. As the proportion of TGF (+) cells increased, cytokeratin-14 was enriched at the invasion boundary, and the highly migratory cell population became behaviorally and molecularly distinct from other cells, which is a factor explaining the formation of leader–follower relationships. Migration mechanisms involving the regulation of cytokeratin-14 alter the physical and chemical properties of cells such as intercellular adhesion and mechanics.

This enhancement did not continue to increase with N; therefore, the second situation was N > 50. Notably, the migration range of TGF (−) did not expand but rather shrank (Figure 4(C3,C4)). Similarly, the migration velocity decreased. Enhanced migration of TGF (+) on TGF (−) is conditional: when the density of TGF (+) is too high, that is, when there are too many leaders, the migration enhancement of followers is inhibited. This causes TGF (−) to lose its centralized leadership, weakening migration enhancement.

The above two situations used N = 50 as the watershed, with the opposite pattern on both sides. A third situation was compared from another perspective to determine the properties that do not have a watershed. We found that straightness decreased with increasing N (Figure 5B), whereas the migration direction moved further away from the fiber with increasing N (Figure 6C). This result indicates that the presence of TGF (+) significantly enhanced the migration of TGF (−). Enhanced migration increases the total distance and thus decreases straightness. Multiple leaders lead to irregularities in the migration direction of their followers.

EMT can be induced by various factors, including transcription factors, growth factors, and microenvironmental miRNAs. We used TGF-β as an induction factor for EMT. TGF-β can induce EMT via signaling pathways such as Smad, RhoA, and MAPK. Our results provide insight into the effects of these processes on cell migration behavior. Cells before and after induction of EMT by TGF-β exhibited different migratory properties and showed interactions at different ratios. To further analyze this phenomenon of cellular interactions, we propose a method based on a combination of time-series clustering and dimensionality reduction to analyze cells with similar migration patterns [26]. Whether cell trajectories are related to migration patterns remains unclear. In the future, this approach will be applied to analyze cell lines with different proportions of phenotypes and has the potential to provide an accurate analysis of interactions based on cell migration trajectories.

## 4. Materials and Methods

### 4.1. Electrospinning

Tetrahydrofuran was used as the solvent in the electrospun polymer solution to prepare 20 wt. % PS. The PS solution was electrospun into aligned nanofiber structures. The polymer solution in the syringe was ejected from the needle at a constant flow rate. A high voltage was applied to the needle, and the charged polymer solution was collected using a grounded rotating collector. During electrospinning, the flow rate was 0.1 mL/h, and collector speed was 15.7 m/s. The electric field was 2.5 kV/cm. The fibers were treated with O_2_ plasma and coated with 10 μg/mL FN [27].

### 4.2. Cell Culture

NMuMG cells (ATCC CRL-1636) and fluorescently labeled NMuMG cells were cultured in Dulbecco’s Modified Eagle Medium (DMEM) supplemented with 10% fetal bovine serum (FBS). Fluorescent labeling of NMuMG was performed by induction of the pDsRed2-C1 vector. All experiments were approved by the ethics committee of the institution. The medium used to induce EMT in NMuMG–DsRed-contained 10 ng/mL TGF-β1 (Sigma) and was cultured for 3 days. Unlabeled NMuMG cells were designated as TGF (−), and EMT-induced NMuMG-DsRed cells were designated as TGF (+). Cell aggregates were prepared by suspending the two cell types at different ratios (1 × 10^5^ cells/mL). The cell suspension (500 μL) was seeded into 24-well grid plates (Elplasia #4445, Kuraray, Tokyo, Japan) for 3D culture and incubated at 37 °C, 5% CO_2_, and 95% humidity for 3 h. FluoroBrite DMEM (Gibco) containing 10% FBS was used for fluorescence observation (Figure 1A). After seeding, cell aggregates formed in the wells. These aggregates were collected and suspended in 500 μL of medium containing 25 μL of CellLight^TM^ Histone 2B-GFP and BacMam 2.0 (Thermo Fisher Scientific K.K., Tokyo, Japan). The aggregates were designated as TGF (N), which represented the percentage of TGF (+) cells (N%). After 24 h of pre-incubation on the fiber sheets, time-lapse images were acquired using a Biostation (Nikon, Tokyo, Japan) at 15 min intervals for 24 h.

### 4.3. Morphological Analysis of Colonies

Fluorescent images of the colonies were binarized and analyzed using Fiji software. The shape of a cell colony was determined by increased area and circularity. Circularity was determined using Equation (1), where *S* and *P* are the area and perimeter of the colony, respectively. A circularity of 1 indicates that the shape is a perfect circle; as the value approaches 0, the shape is considered to have elongated.

(1) $$\begin{array}{c} Circularity=4\pi \times \frac{S}{P^{2}} \end{array}$$

The definition of the center of mass of a colony was determined using Equation (2). This value represents the average of the coordinates of n cells in the collective at a certain time. The initial position was set to (0,0).

(2) $$\begin{array}{c} Center\;of\;mass=\frac{1}{n}\overset{n}{\underset{i=1}{\sum }}\left(x_{i},y_{i}\right) \end{array}$$

### 4.4. Migratory Analysis of Single Cell

Fluorescent images of cell nuclei were binarized and analyzed using the Fiji plug-in (TrackMate) to extract the coordinates of motion of each cell at each time point and trajectory. The velocity, distance, directionality, and angle of cell migration were calculated using these coordinates. The straightness was determined using Equation (3) as the ratio of the Euclidean distance of cell migration to the total distance.

(3) $$\begin{array}{c} Straightness=\frac{d_{Euclid}}{d_{Total}} \end{array}$$

## 5. Conclusions

We established a method for observing the migration of cell colonies on fibers. Using this method, we observed the migration of colonies using different ratios of TGF (+) and TGF (−) to quantify the effect of TGF (+) on collective migration. Migration was assessed from two perspectives: that of the entire colony and that of each cell within the colony. When TGF (+) was present at low densities in the colony, the migration distance of individual cells and behavior of the colony were significantly enhanced. This enhancement decreased when the TGF (+) density exceeded a certain level. These collective behaviors were caused by a leader–follower-like structure. We analyzed individual cells during collective migration. In the future, by precisely analyzing the interactions among individual cells, it will be possible to assess the migratory properties of collective migration more clearly.

## Data Availability

The datasets generated and/or analyzed during the current study are available from the corresponding author upon reasonable request.

## Supplementary Materials

The following supporting information can be downloaded at: https://www.mdpi.com/article/10.3390/ijms232012372/s1.
